# A Systematic Review of Tai Chi-based Interventions for Positive and Negative Symptoms, Cognitive Functioning, and Quality of Life in Psychosis

**DOI:** 10.1007/s10597-025-01483-8

**Published:** 2025-07-21

**Authors:** Donagh Seaver O’Leary, David Marshall, Justin Smyth, Keith Gaynor, Mary Clarke

**Affiliations:** 1 School of Psychology, University College Dublin, Dublin, Ireland; 2 DETECT, Early Intervention Service, Blackrock, Co. Dublin Ireland; 3 St. John of God Hospitaller Services Group, Stillorgan, Co. Dublin Ireland; 4 School of Medicine, University College Dublin, Dublin, Ireland

**Keywords:** Tai Chi, Qigong, Psychosis, Schizophrenia, Mind–body Intervention

## Abstract

This study aimed to provide a systematic review of Tai Chi interventions in psychosis and their impact on positive and negative symptoms, cognitive deficits and quality of life. There is growing evidence that the mind–body practice of Tai Chi has value in treating and preventing mental health-related problems, such as stress and anxiety. However, it is unclear whether they are effective or beneficial in serious mental illnesses such as psychosis. A systematic review was conducted in accordance with PRISMA guidelines. The review protocol was registered (PROSPERO). The review examined studies which included adults with diagnosed psychotic disorders who participated in treatment studies examining Tai Chi or Qigong interventions using quantitative measures of positive and negative symptoms, cognitive deficits and quality of life. The search was conducted in August 2024. Data were extracted by two independent researchers and analysed using a narrative synthesis approach. Three-hundred and eighty one papers were screened with six studies included in the final review. Publications spanned from 2012–2022. All studies were based in Asia. Tai Chi interventions were acceptable, and well-tolerated. Outcomes indicated small-medium effective sizes in improving negative symptoms, and cognitive deficits. However largely, these improvements were not maintained over time. The current review suggests that Tai Chi is an acceptable, well-tolerated intervention in individuals with long-term psychotic illnesses in Asian populations There is tentative evidence that it may have a positive impact for negative symptoms and cognitive deficits. Well-controlled studies should be encouraged in Western settings.

**Supplementary Information:**

The online version contains supplementary material available at 10.1007/s10597-025-01483-8.

## Introduction

**Fig. 1:**
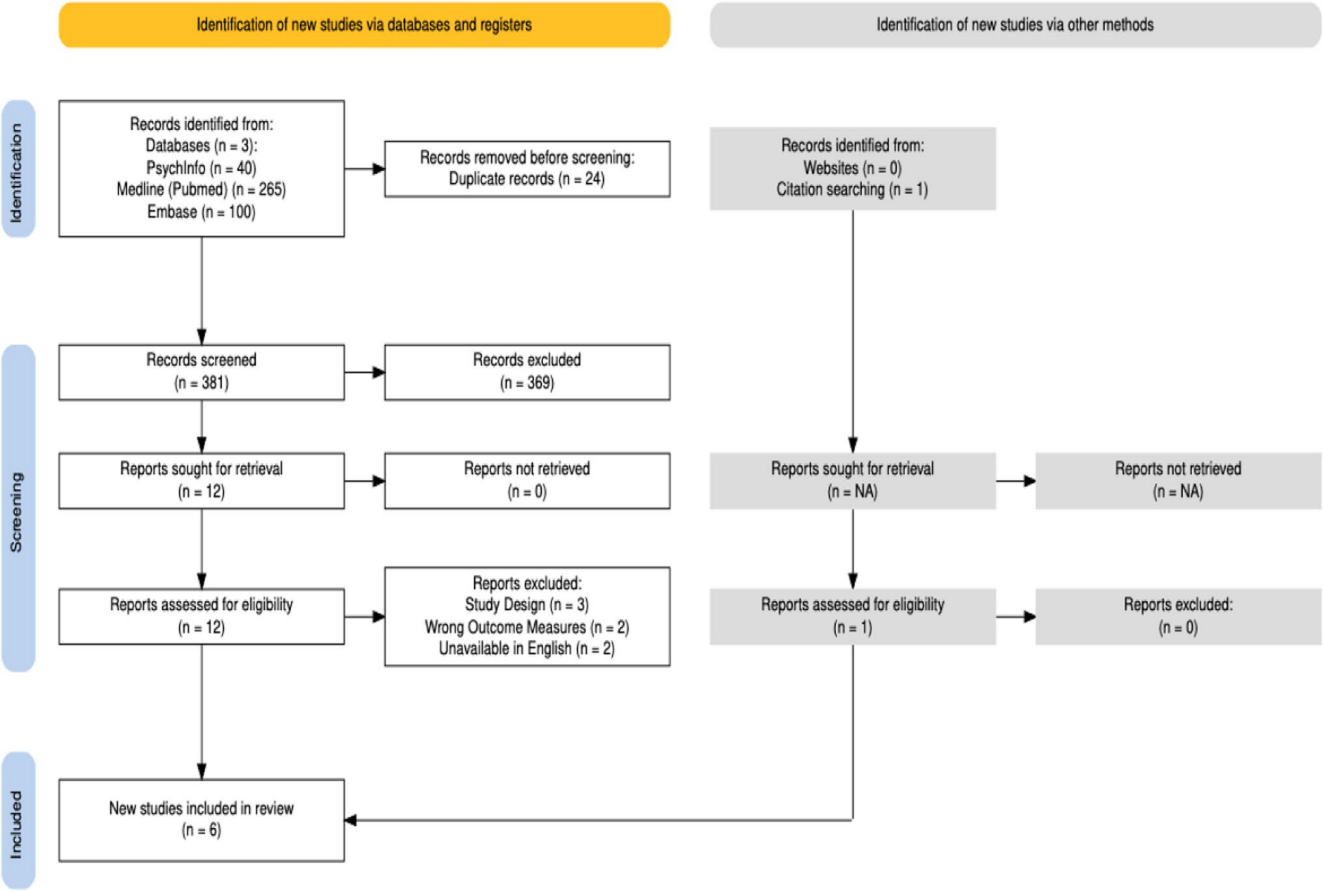
Prisma diagram

The Global Burden of Disease ranks psychosis as one of the most disabling medical conditions globally (Charlson et al., 2018). The symptoms of psychosis include positive symptoms such as hallucinations and delusions, negative symptoms such as avolition, anhedonia, social withdrawal, alogia and affective flattening (Bègue et al., 2020; Tandon et al., 2024), cognitive dysfunction (e.g., Sheffield et al., 2018). Furthermore, it results in reduced quality of life, social functioning and occupational functioning (e.g., Cowman et al., 2024; Hoseinipalangi et al., 2022) for many people (Fig. 1).

### Unmet Therapeutic Needs in Psychosis

The evidence-base for the effective treatment of positive symptoms of psychosis is relatively strong (e.g., Barnes et al., 2020). However, negative symptoms are currently recognised as an unmet therapeutic need in a large proportion of individuals and are a significant source of functional impairment (Kirkpatrick et al., 2006). This may be related to the relative ineffectiveness of most pharmacological treatments—apart from Clozapine– to improve negative symptoms (Aleman et al., 2017; Correll & Schooler, 2020). Similarly, cognitive deficits related to psychosis lead to significant social and occupational impairment (Montaner-Ferrer et al., 2023) but very often go under-treated despite evidence of effective interventions (Allott et al., 2020). Furthermore, the physical health burden associated with psychotic illness is substantial (e.g., Foley et al., 2013; Stubbs et al., 2016).

Apart from medication, other evidence based interventions routinely offered include cognitive behavioural therapy (CBT), family psychoeducation, and occupational support. However, despite the availability of these interventions, only one third of those with psychosis will go on to achieve full functional recovery, and quality of life can remain impaired for many. In a recently published longitudinal study by O’Keeffe and colleagues (O’Keeffe et al., 2022), 50% of the sample showed reduced QOL scores at 20-year follow up following first episode psychosis (FEP). Those with psychosis have a low uptake of available psychological treatments, especially talk therapies such as Cognitive Behavioural Therapy (e.g., Gaynor et al., 2014). Evidence also shows a high level of disengagement from services, with rates of disengagement consistently found to be around 33% (Doyle et al., 2014). Given this, there is a clear unmet need to offer alternative, empirically supported therapeutic interventions that engage a wider selection of the FEP cohort.

### Mind–Body Therapies and Psychosis

There has been increasing global interest in mind–body therapies to address the rising incidence of mental health conditions (Goldberg et al., 2018; Vancampfort et al., 2021). Resultingly, several professional organisations have recently included the use of mind–body therapies as adjunct interventions in their recommendations for best practice, including the UK National Institute for Health and Care Excellence (National Institute and for Health and Care Excellence, 2018) and the Canadian Network for Mood and Anxiety Treatments (Ravindran et al., 2016). Recently, a series of systematic reviews have highlighted early evidence for the effectiveness of mind–body therapies such as Yoga, Tai-Chi and Qigong for symptoms of psychosis (Martinez-Calderon et al., 2024; Su et al., 2024; Wei et al., 2020; Yip et al., 2022). The evidence points towards their impact on negative symptoms, cognitive deficits and improved quality of life. However, it is noticeable that many of these reviews conflate different mind–body therapies, exercise interventions, and mindfulness interventions, so services may struggle to identify specific interventions to offer clients (Rißmayer et al., 2024). Similarly, these approaches draw on very different theoretical underpinnings, have varying intervention formats, and have been tested with a range of methodologies, making it hard to highlight what the effective components are and what adaptations are needed (Rißmayer et al., 2024; Su et al., 2024). Additionally, many studies test only one symptom profile e.g., negative symptoms, leaving knowledge gaps in relation to other areas of dysfunction.

### Tai Chi and Qigong

Qigong is a combination of qi (aka life-force, life energy, bioenergy, creativity, consciousness, breath, function) and gong (cultivation or practice over time, as in the practice of an Art). Qigong is a self-initiated health and wellness practice consisting of a combination of exercise (posture, movement, and breathing techniques) and meditation (mindfulness and interoception). Researchers describe Qigong as a meditative movement that has the following characteristics: (a) some form of movement or body positioning, (b) a focus on breathing, and (c) a cleared or calm state of mind with a goal of (d) deep states of relaxation (Larkey et al., 2009). Tai Chi originated as an ancient martial art, but over the years it has become more focused on health promotion and rehabilitation. When Tai Chi is performed for health, it is considered a form of Qigong and involves integrated physical postures, focused attention, and controlled breathing. Tai Chi is one of the hundreds of forms of Qigong exercises that were developed in China. Other forms of Qigong include Baduanjin, Liuzijue, Hu Yue Xian, Yijin Jing, and medical Qigong (Lo et al., 1979).

There is growing evidence that the mind–body practice of Tai Chi/Qigong (abbreviated to Tai Chi) has value in treating and preventing many health-related problems, such as stress and anxiety, and that regular practice helps to significantly improve well-being, attention, focus, and resilience (Lee & Ernst, 2012; Lee et al., 2011; Sharma & Haider, 2015; Wang et al., 2014). Tai Chi has also been used in the treatment of depression (Yin et al., 2023).

### Research Aims

Following on from the recommendations of Martinez-Calderon and colleagues (Martinez-Calderon et al., 2024), this review examines a Tai Chi specifically, separately from other mind–body practices, focusing on the impact on symptoms of psychosis; positive and negative symptoms, as well as cognitive deficits and quality of life. Utilising systematic review guidelines, the current study aims to provide a systematic review of the literature related to the therapeutic use of Tai Chi and psychosis.

## Methods

### Registration

The review protocol was conducted in accordance with the Preferred Reporting Items for Systematic Reviews and Meta-analyses (PRISMA) guidelines (Moher et al., 2009).

### Search Strings and Databases

Searches were conducted in Psychinfo, Medline (Pubmed) and Embase in August 2024. The following search string criteria was used:

$$\begin{array}{c}\lbrack(((\mathrm{tai}\;\mathrm{chi})\;\mathrm{OR}\;(\mathrm{tai}-\mathrm{chi}))\;\mathrm{OR}\;((\mathrm{qigong}))\;\mathrm{OR}\;(\mathrm{qi}-\mathrm{gong})))\;\\\mathrm{AND}\;((\mathrm{schizophrenia})\;\mathrm{OR}\;(\mathrm{psychosis}))\rbrack\end{array}$$

Please see Supplemental Material 1 for extra search strings. The Qigong Institute database was also utilised. However, the limited search engine capacities meant that searches were manual and limited to variations of ‘Tai Chi’ ‘Qi Gong’ ‘Psychosis’ and ‘Schizophrenia’. Finally, additional records were identified by researchers via manual searches.

### Inclusion and Exclusion Criteria

A full list of the PICO criteria can be seen in Table 1. To be included in the analysis, studies had to fulfil the following inclusion and exclusion criteria. Inclusion criteria included: (1) Studies of people with psychosis (defined by a diagnosis of a primary psychotic disorder based on DSM or ICD criteria OR scores above clinical cut-off for positive or negative symptoms of psychosis on validated clinical instruments). (2) Controlled or uncontrolled treatment studies that include quantitative outcome data derived from psychometrically validated measures. (3) Studies examining Tai Chi or Qigong interventions. Exclusion criteria included (1) Studies with non-clinical samples. (2) Studies that do not include psychometrically validated quantitative outcome data. (3) Studies not in English or not translated into English. (4) Studies not in peer reviewed journals. (5) Studies prior to 1985.

**Table 1:** PICO criteria for study selection

Population	Adults with a clinical diagnosis of a psychotic disorder
Intervention	A formally defined Tai Chi or Qi Gong intervention
Comparison	Control intervention: waitlist, TAU or Pre/Post Baseline
Outcome	Change in positive or negative symptoms, cognitive function or quality of life, including adverse outcomes

### Data Extraction

All papers were downloaded from each database onto Zotero, a reference management software. From Zotero they were uploaded to Covidence, an online software tool for conducting systematic reviews. Using Covidence, data were screened for applicability by two independent reviewers. Any disagreement occurring were arbitrated by a third reviewer, the principal investigator (KG). A standardised extraction form was designed on Microsoft Excel to extract data from the chosen studies on aspects, such as demographics, study design, population, interventions and comparators, outcome measurements, clinical outcomes, and information for the assessment of the risk of bias.

### Quality Assessment

Study quality was determined using The Evidence Project Risk of Bias tool for systematic reviews (Kennedy et al., 2019). The tool includes eight items, each of which is rated as being present (yes) or not present (no) and, for some items, not applicable or not reported (Table 2). The items include (1) Cohort (Does this study include a cohort that completed both the baseline assessment and the follow-up assessment?); (2) control or comparison group (Does this study present data from both before (baseline) and after the intervention?); (3) pre-post intervention data, (4) randomisation (Were participants randomly assigned to intervention and/or control arms?); (5) random selection of participants for assessment, (6) follow-up rate of 80% or more, (7) comparison groups equivalent on sociodemographic measures, (8) comparison groups equivalent at baseline on outcome measures, and (9) comparison groups equivalent at baseline on outcome measures. Together, items (1) to (3) summarize the study design, while the remaining items consider other common elements of study rigor.

**Table 2:** Risk of bias across studies

Author (year)	Cohort	Control or Comparison	Pre/Post	Randomisation	Random Selection	Follow up of 80%	Comparison 1	Comparison 2	Comparison 3
Ho et al. (2012)	y	y	y	y	n (purposeful sampling)	y (20% attrition rate)	y	y	y
Ho et al. (2016)	y	y	y	y	n (purposeful sampling)	y	y	y	y
Kang et al. (2016)	y	y	y	y	n (purposeful sampling)	y	y	y	y
Li et al. (2020)	y	y	y	y	n (purposeful sampling)	n/a (attrition not reported)	y	y	y
Gao et al. (2021)	y	y	y	y	n (purposeful sampling)	y	y	y	y
Chen et al. (2022)	y	y	y	y	n (purposeful sampling)	y	y	y	y

(1) Cohort (Does this study include a cohort that completed both the baseline assessment and the follow-up assessment?); (2) Control or Comparison (Does this study present data from both before and after the intervention?); (3) Pre/Post (Does this study have a control and/or comparison arm in addition to the intervention arm?); (4) Randomisation (Were participants randomly assigned to intervention and/or control arms?); (5) Random Selection (Were participants randomly selected for assessment?), (6) Follow-up rate 80% (Was there a follow up rate of at least 80%); (7) Comparison 1 (Were comparison groups equivalent on sociodemographic measures?); (8) Comparison 2 (Were comparison groups equivalent on severity of illness measures?); (9) Comparison 3 (Were comparison groups equivalent at baseline on outcome measures?). y = yes, *n* = no

### Data Synthesis

A narrative synthesis (Popay, 2006) was chosen as the method of data analysis due to the heterogeneity of included studies in relation to the sample population, study design, context, and data analyses utilised. This review followed the general framework of narrative synthesis, as outlined in the guidance documents supporting this synthesis (Popay, 2006). This includes: (i) an initial descriptive summary and explanation of the characteristics and findings of each included study, (ii) a preliminary synthesis of findings of included studies, (iii) an exploration of relationships and finding consistencies within and between studies, and (iv) an assessment of the robustness of this synthesis.

## Results

### Study Selection

Database searches yielded 405 references (PsychInfo = 40; Medline (Pubmed) = 265; Embase = 100) which were imported into Covidence. After duplicates were removed, 381 papers remained for Title and Abstract screening. Ineligibility led to a further 369 exclusions.

12 papers were subject to full-text reviews. An additional paper was sourced by manual searching. No extra papers were sourced from The Qigong Institute database. Six papers were included for the final data extraction phase.

## Principal Findings

### Qualitative Description of Included Studies

Six studies in total were included in this review. Publications spanned from 2012–2022. All were based in Asia (2 from Hong Kong, 3 from China, and 1 from Taiwan). The intervention modality used included Tai Chi (*n* = 3) (Ho et al., 2012, 2016; Kang et al., 2016), Baduanjin (*n* = 2) (Chen et al., 2022; Li et al., 2020) and Yijinjin (*n* = 1) Gao et al., (2021). Kang et al., (2016) Tai Chi group received simultaneous social skills training.

Over the 6 studies, 240 intervention participants and 318 controls were included. The type of control groups used varied. Ho et al., (2012), Kang et al., (2016) and Gao et al., (2021) used waitlist controls receiving treatment as usual (TAU). Ho et al., (2016) used a three arm design with the first control being a TAU waitlist and the second comprising of an aerobic exercise intervention specifically designed to match the VO2 expenditure of Tai Chi. Both Li et al., (2020) and Chen et al., (2022) used a ‘brisk walking’ control matched to time spent doing Baduanjin.

All studies’ participants were diagnosed with schizophrenia, either by DSM or ICD criteria, and receiving stable medication dosages. Participants across all included studies were excluded if they were (a) suffering from severe acute symptoms, (b) had any physical disability (cardiovascular, musculoskeletal or pulmonary), and (c) had pronounced visual or auditory impairments. Some studies excluded potential participants if they had intellectual disabilities (Chen et al., 2022; Gao et al., 2021; Ho et al., 2016; Li et al., 2020). Although the age range was 18–69, participants were predominantly middle age and with a course of illness in terms of decades (Table 3).

**Table 3:** Characteristics of participants across studies

Lead Author, year (Country)	Participants (*N*)	Control Type (*N*)	Age *M (SD*)	Illness Duration in years *M* (*SD*)	Hospitalisation in years (*M*)	Diagnosis	Intervention Group Baseline Symptom Severity (PANSS, PANSS subscales) *M* (*SD*)	Control Group Baseline Symptom Severity (PANSS, PANSS subscales) *M* (*SD*)
Ho, Rainbow T.H., 2012 (Hong Kong)	15	TAU (15)	51.87 (10.85)	ca. 28 (n/a)	11.8	Schizophrenia (DSM IV-TR)	*SANS subscales Attention: 4.27 (3.39) Anhedonia-asociality: 4.53 (4.29) Avolition-apathy: 1.67 (2.82) Alogia: 4.4 (4.97) Affective blunting: 8.2 (7.98)	*SANS subscales Attention: 5.6 (3.81) Anhedonia-asociality: 4.4 (4.6) Avolition-apathy: 2.27 (3.37) Alogia: 4.8 (5.17) Affective blunting: 8.67 (7.76)
Ho, Rainbow T.H., 2016 (Hong Kong)	51	Exercise (51), Waitlist (51)	52.4 (9.6)	28.5 (9.8)	n/a	Schizophrenia (DSM IV-TR)	Positive**:** 6.4 (2.9) Negative: 12.4 (5.6) Excitement: 5.0 (1.9) Depression: 5.3 (2.7) Cognitive: 3.9 (2.5)	Positive: 7.6 (3.6) Negative: 13.3 (5.4) Excitement: 6.2 (2.7) Depression: 6.0 (2.8) Cognitive: 4.4 (2.6)
Kang, Ruiying, 2016 (China)	118	TAU (126)	45.9 (12.1)	21.3 ± 11.7	n/a	Schizophrenia, (ICD-10)	PANSS Total: 44.5 (3) Positive: 10.1 (1.4) Negative: 13.4 (1.5) General Psychopathology: 22.0 (2.0)	PANSS Total: 45.2 (3) Positive: 10.2 (1.6) Negative: 13.6 (1.5) General Psychopathology: 21.7 (2.0)
Li, Mingli, 2020 (China)	30	Brisk Walking (30)	51 (6.86)	21.63(10.13)	n/a	Schizophrenia (DSM IV)	PANSS Total: 61.67 (11.38)	PANSS Total: 62.58(14.42)
Chen, Chyi-Rong, 2022 (Taiwan)	24	Brisk Walking (30)	50.63 (6.11)	27.25 (7.75)	5.17 ± 3.46	Schizophrenia (DSM V)	n/a	n/a
Gao, Hui, 2021 (China)	20	TAU (20)	53.40 (7.04)	31.75 ± 5.31	12.65 ± 6.48	Schizophrenia (ICD-10)	PANSS Total: 69.45 (13.57) Positive: 12.65 (4.52) Negative 24.35 (5.18) General psychopathology: 32.45 (6.83)	PANSS Total: 67.8 (15.08) Positive: 12.7 (6.11) Negative 22.1 (4.8) General psychopathology: 33.0 (7.45)

*TAU*, Treatment as Usual; *PANSS*, Positive and Negative Symptoms Scale; *SANS*, Scale for Assessment of Negative Symptoms

Intervention durations varied between 6 weeks (Ho et al., 2012), 12 weeks (Chen et al., 2022; Gao et al., 2021; Ho et al., 2016; Kang et al., 2016), 24 weeks (Li et al., 2020) and 12 months (Kang et al., 2016). Excluding Gao et al., (2021) all interventions were administered by health care professionals such as nurses, psychologists, psychiatrists who had received specialised training. Frequency of intervention ranged considerably from 45 min every 2 weeks to 200 min per week (Table 4).

**Table 4:** Characteristics of included studies

Lead Author, year (Country)	Modality	Study Design	Duration	Frequency	Pos Sym. Measure	Neg sym Measure	Cog Sym Measure	QOL sym Measure
Ho, Rainbow T.H., 2012 (Hong Kong)	Tai Chi	RCT	6 weeks	150 min/week	n/a	SANS	CMDT	WHODAS-II
Ho, Rainbow T.H., 2016 (Hong Kong)	Tai Chi	RCT	12 weeks	150 min/week	PANSS	PANSS	WAIS, NES	ADL IADL
Kang, Ruiying, 2016 (China)	Tai Chi alongside Social Skills Training	RCT	12 months	45 min/2 weeks	PANSS	PANSS	n/a	WHOQOL-BRIEF
Li, Mingli, 2020 (China)	Baduanjin	RCT	24 weeks	200 min/week	PANSS	PANSS	WMS, TMT, DSST	n/a
Chen, Chyi-Rong, 2022 (Taiwan)	Baduanjin	RCT	12 weeks	80 min/week	n/a	n/a	MoCA, TMT, WMS	ADLRS- III
Gao, Hui, 2021 (China)	Yijinjing	RCT	12 weeks	120 min/week	PANSS	PANSS	MMSE	ITAQ

*SANS*, Scale for the Assessment of Negative Symptoms; *PANSS*, Positive and Negative Symptoms Scale; *CMDT*, Minnesota Rate of Manipulation Test; *WAIS*, Wechsler Adult Intelligence Scale; *NES*, Neurological Evaluation Scale; *WMS*, Wechsler Memory Scale; *TMT*, Trail Making Test; *MoCA*, Montreal Cognitive Assessment; *DSST*, Digit Symbol Substitution Test; *MMSE*, Mini Mental State Examination; *WHODAS-II*, World Health Organization Disability Assessment (partly modified for Chinese language and participant's lifestyle); *ADL*, Barthel's Activities of Daily Living index; *IADL*, Lawton's Instrumental Activities of Daily Living; *WHOQOL-BRIEF*, World Health Organization Quality of Life (Brief); *ADLRS- III*, Activities of daily living rating scale III; *ITAQ*, Insight and Treatment Attitude Questionnaire

### Heterogeneity

Comparing the included studies, there was a large heterogeneity evident for intervention type, intervention length and duration, and the type of control used. The different modalities (Tai Chi, Baduanjin and Yininjing), although broadly similar, are distinct practices. Studies were largely homogenous in terms of population demographics used including key features such as type of diagnosis, duration of illness, baseline symptom severity measures, age, and the geographical location of the study (Table 3). All studies reported no statistically significant group differences in baseline scores across all sociodemographic variables, assessment variables and illness severity measures. The exclusion criteria for participation did not notably differ across studies.

### Symptom Outcomes

#### Positive Symptoms Results

Four out of the 6 studies included measurements for Positive Symptoms (Gao et al., 2021; Ho et al., 2012, 2016; Li et al., 2020), using the Positive and Negative Symptoms Scale (PANSS; Table 4). No study reported significant findings for positive symptom outcomes.

#### Negative Symptoms

Except for Chen et al., (2022), all of the papers included measurement for negative symptoms. Four papers used the PANSS to measure negative symptoms (Gao et al., 2021; Ho et al., 2016; Kang et al., 2016; Li et al., 2020). Ho et al., (2012) pilot study used the Scale for the Assessment of Negative Symptoms (SANS).

Kang et al. (Kang et al., 2016) and Gao et al., (2021) both reported statistically significant improvements in negative symptoms. Kang et al., (2016) found small but significant improvement for the Tai Chi intervention over time and compared to the control group, both at the halfway point (*t* = 6.472, *p* < 0.001) and endpoint of their 12 month intervention (*t* = 8.250, *p* < 0.001 Gao et al., (2021) reported improvements for the Tai Chi intervention compared to baseline in the negative subscale (*t* = 19.00, *p* < 0.0001), general psychopathology subscale (*t* = 15.98, *p* < 0.0001), and total score (*t* = 15.47, *p* < 0.0001). Compared to the respective scores in the control group, the negative subscale score significantly improved (*t* = 2.953, *p* = 0.0054). No effect sizes were reported in either paper.

#### Cognitive Symptoms

Of the 5 studies that included cognitive assessment, a range of cognitive modalities were assessed. Specifically, memory was measured in Ho et al., (2016), Li et al., (2020) and Chen et al., (2022). Motor coordination was assessed in Ho et al. (Ho et al., 2012), Ho et al., (2016) and Li et al., (2020), and working memory by Ho et al. (Ho et al., 2016), and Li et al., (2020). More generalised cognitive batteries were used by Gao et al., (2021; Chen et al., 2022).

Improvement in cognitive symptoms represented the most consistent finding across papers. Gao et al. (Gao et al., 2021) found small improvements for both the intervention and control groups in generalised cognition, with greater improvements for the intervention group compared to controls (*t* = 2.2.68, *p* = 0.0291). Chen et al., (2022) intervention group showed small improvements in global cognitive function across multiple domain measures immediately after intervention, but the effect was not maintained at the follow-up assessment. Li et al., (2020) presented the most striking results, showing around 50% improvement in memory testing over the course of the Tai Chi intervention compared to a matched brisk walking control with a moderate effect size (F = 6.21, *p* = 0.003, ɳ^2^ = 0.095). Despite finding cognitive improvement in their pilot study, in the follow up study, Ho et al., (2016) found no significant difference between Tai Chi and exercise intervention on any cognitive modality.

#### Quality of Life

Every paper except Li et al., (2020) included a quality of life measurement. Measurement was largely heterogeneous. Ho et al., (2012) and Kang et al., (2016) focused on general quality of life. Ho et al., (2016) and Chen et al., (2022) general daily functioning. Gao et al., (2021) used an insight and treatment attitude measure.

Neither Ho et al., (2016) or and Chen et al., (2022) found any significant findings for quality of life measures. Ho et al., (2012) reported small improvements in interpersonal functioning domains (*p* = 0*.*01). Kang et al., (2016) reported improvement in psychological quality of life in the social domain (*t* =–2.171, *p* = 0.031). Effect sizes were unreported. Interestingly, Gao et al., (2021) found a small steady decrease in self-esteem over the course of intervention (*p* < 0.05) but this change was not statistically significant when compared to the control group.

#### Adverse Effects

Ho et al., (2012) pilot study included a qualitative interview post-intervention, where participants offered feedback on the difficulties that arose during the Tai Chi trial. Authors combined their answers into the following categories: (1) The experience of tiredness; (2) The resulting bodily discomfort during practise; (3) The difficulty executing and remembering of the Tai-chi movements; (4) The difficulty in practising independently; (5) Tai Chi being slow, mundane and boring. No other study identified any adverse effects.

Ho et al., (2016) analysed saliva samples at 4 points across the day to measure stress levels during the Tai Chi intervention. Intervention participants experienced an increase in mean cortisol levels, but this dissipated at follow-up.

## Discussion

### Main Findings

Prompted by Martinez-Calderon et al., (2024), this systematic review examined whether Tai Chi interventions were effective in improving positive and negative symptoms, quality of life, or cognitive functioning in people with psychosis. Although recent systematic reviews have included Tai Chi as a subdomain of more general mind–body interventions (Rißmayer et al., 2024; Sabe et al., 2019; Su et al., 2024) none since Zheng et al., (Zheng et al., 2016) have examined Tai Chi’s impact on symptoms of psychosis specifically. The included studies showed a wide variety of types, frequency and duration of Tai Chi interventions. Thus, it is difficult to arrive at any definitive conclusion about the efficacy of intervention in the context of such heterogeneity. However, some clear, albeit tentative, findings can be considered. Tai Chi showed some promise in improving negative symptoms, ameliorating some cognitive symptoms and improving some quality of life measures in individuals with long term psychotic illnesses in Asian populations. Given the small to moderate effect sizes and large heterogeneity between studies, these results should be interpreted with caution and taken as preliminary.

Although participants discussed tiredness and boredom as issues in Tai Chi practice, there was no evidence of adverse effects or a deterioration of symptoms due to Tai Chi, indicating its safety and applicability. We noted that there were very few dropouts reported across included studies. Both Kang et al., (2016) and Gao et al., (2021) reported a 0% attrition rate in their studies. Ho et al., (2016) reported eight total dropouts (5.2% attrition rate), however only one of these was from the Tai-Chi intervention group. Chen et al., (2022) reported one dropout from the intervention group (4% attrition rate) noting that this was due to relocation. The most dropouts was reported in Ho et al., (2012) pilot study, with a total of seven (20% attrition rate). However, all seven participants completed the intervention and were lost at the follow up stage due to “physical illness or relapse leading to hospitalisation” (Ho et al., 2012), p.5. Only Li et al., (2020) did not provide information on attrition rates. This low attrition rate is indicative of Tai Chi being an easy to administer intervention. However, Ho et al., (2012) post-intervention qualitative feedback provide some interesting clues as to important adaptations to be made to interventions to maintain attention, and engagement and to minimise tiredness. The negative symptoms common to those with schizophrenia include pervasive anhedonia and avolition. For this population, daily functioning tasks are already difficult, and the addition of a Tai Chi practice may be too much to take on without requisite support from caregivers. Furthermore, complaints of “*difficulty executing and remembering the Tai Chi movements*” and “*difficulty in practicing independently*” constitute a conspicuous barrier to those with marked cognitive impairments common to many people with long durations of the condition. These findings highlight the importance of acceptability and qualitatively designed studies in this area to appropriately design interventions.

Cautiously, we can conclude that there is some preliminary evidence that Tai Chi interventions may improve negative symptoms. When reported, the effect sizes were in the small to moderate range, which may make these results to be of questionable clinical relevance. Nevertheless, it is important to note that negative symptoms are often enduring and difficult to treat. Thus, even small improvements in symptoms might be relevant to those patients. In line with Zheng et al., (2016), we found no effect of Tai Chi on positive symptoms.

Broadly, the results for improvements in cognitive deficits are considered too heterogenous to determine any substantive finding. Nonetheless, the results are indicative of a potential buffering effect of the degenerative course of cognition in those living with long-term psychosis (Tandon et al., 2024). Continued Tai Chi interventions may be suitable intervention for slowing such neurocognitive deterioration. However, the *ongoing* nature of such interventions appears a crucial factor, as some of our reviewed studies reported that effects were not maintained in post-intervention follow-ups.

Although some indications of improvements in quality of life measures were reported, they are broadly too heterogenous in their measures to arrive at any certain conclusion at this time. Further research is needed.

## Active Therapeutic Ingredients

Rißmayer et al., (2024) highlighted that key factors in successful mind–body interventions are similar to psychosocial interventions in general: the quality of the instructor, the specificity of the intervention and how well the sample is matched to the intervention. All except one study (Gao et al., 2021) used trained mental health practitioners (e.g., nurses, occupational therapists and psychology assistants) to administer interventions. This is potentially a significant limitation and highly trained instructors in both Tai Chi and mental health may be necessary to deliver a theoretically coherent intervention. For instance, within the mindfulness literature, the experience and consistent practice of the instructor has a demonstrable effect on the client outcome. Additionally, we agree with Rißmayer et al., (2024) contention that intensity of intervention is a crucial factor in standardising studies. Across our studies, intensity varied widely, from 45 min every 2 weeks to 200 min every week.

## Limitations in the Literature

Several studies exist outside English language publications (Zheng et al., 2016). This has previously been commented upon by non-Asian researchers (Vogel et al., 2019), and is a conspicuous gap in the possible range of our included studies. Heterogeneity is the most common limitation reported across reviews, making the comparison of cognitive and quality of life measures and different interventions challenging. Unfortunately, in many of the included studies, the strength of evidence was not reported in terms of effect sizes. Relatedly, Sabe et al., (2019) review specifically excluded Kang et al., (2016) paper that combined social skills training and Tai Chi “*because social skills training is one of the few evidence- based interventions for negative symptoms and the combination treatment thus precludes the identification of specific tai-chi effects*” (Sabe et al., 2019), p. 17.

All studies included this review took place in Asia raising the question as to how comparable studies would work in non-Asian settings. This is important because the foundations of Tai Chi interventions are anchored in Asian culture. It is therefore an open question whether the present results translate to Western countries at all. The concepts underpinning Qigong are fundamental to Chinese medicine and would be well understood by the community at large. In contrast, Tai Chi in the West is a niche interest and is often undertaken as an exercise separate from its theoretical grounding in Chinese medicine.

## Strengths and Limitations

This study has a number of strengths, Firstly, the specificity of the research question, the use of multiple databases, the validity of including a second reviewer in the screening process, and the inclusion of a manual search of the Qigong Institute database. Secondly, we manually combed through all of the other available recent Systematic Reviews and Meta-Analyses in the domain of Mind–Body exercise and psychosis. Our Systematic Review includes every paper mentioned in 8 similar publications between 2016–2024 (Li et al., 2018; Rißmayer et al., 2024; Sabe et al., 2019; Su et al., 2024; Vogel et al., 2019; Wei et al., 2020; Yip et al., 2022; Zheng et al., 2016). Many include studies including mind–body exercises such as Tai Chi, Baduanjin and Yijinjing; the majority, however, are focused on yoga.

It is a significant limitation of the study that only English language research could be included, especially considering all studies were of Asian origin and Tai Chi being a geographically and culturally specific intervention.

### Future Directions

We believe that further studies investigating the efficacy of Tai Chi for people with psychosis is justified. These findings highlight the importance of tailoring interventions based on acceptability to participants, and co-designed studies in this area will be of value to appropriately aid such progress. Reducing heterogeneity in cognitive and QoL assessments would allow for greater comparison between studies. Further studies could explore which particular sub-groups might benefit from a Tai Chi intervention, whether specific forms Tai Chi may have greater benefit, as well as examining the issue of the number and frequency of sessions.

## Conclusion

The current review suggests that Tai Chi is an acceptable, well tolerated intervention in people with psychosis. There is tentative evidence that it may have a positive impact for negative and cognitive symptoms. Well-controlled studies should be encouraged in Western settings, emphasising the importance of therapist factors, examining a broad range of health outcomes.

## Supplementary Information

Below is the link to the electronic supplementary material.

Supplementary file1 (DOCX 22 KB)

## Declarations

### Trial Registration

NA
