# Supplementary material for: A Systematic Review of Tai Chi-based Interventions for Positive and Negative Symptoms, Cognitive Functioning, and Quality of Life in Psychosis
Source: Community Ment Health J. 2025 Jul 21;61(8):1481–91. doi: 10.1007/s10597-025-01483-8 (PMC12647228; doi:10.1007/s10597-025-01483-8)
Supplement: Supplementary file 1 — Supplementary file1 (DOCX 22 KB) [file 10597_2025_1483_MOESM1_ESM.docx]

**Supplemental Material 1**

After initial exploratory searches, due to noticeable syntax differences between the three databases, the following permutations for each were used:

**Psychinfo:** ((tai-chi) OR ('tai chi') OR (qi-gong) OR (qigong)) AND ((schizophrenia) OR (psychosis))

**Medline(Pubmed):** ("Physical Therapy Modalities"[MESH]) AND (psychosis)

**Embase:** ('tai chi' OR 'qi gong' OR qigong) AND ('schizophrenia'/exp OR schizophrenia OR 'psychosis'/exp OR psychosis)
